# Double Selection Enhances the Efficiency of Target-AID and Cas9-Based Genome Editing in Yeast

**DOI:** 10.1534/g3.118.200461

**Published:** 2018-08-15

**Authors:** Philippe C Després, Alexandre K Dubé, Lou Nielly-Thibault, Nozomu Yachie, Christian R Landry

**Affiliations:** * Département de Biochimie, Microbiologie et Bio-informatique, Faculté de sciences et genie, Université Laval, Québec, Québec, G1V 0A6, Canada; † PROTEO, The Québec Research Network on Protein Function, Structure and Engineering, Université Laval, Québec, Québec, G1V 0A6, Canada; ‡ Centre de Recherche en Données Massives (CRDM), Université Laval, Québec, Québec, G1V 0A6, Canada; § Département de Biologie, Faculté de sciences et Génie, Université Laval, Québec, Québec, G1V 0A6, Canada; ** Research Center for Advanced Science and Technology, Synthetic Biology Division, University of Tokyo, Tokyo, 4-6-1 Komaba, Meguro-ku, 153-8904, Japan; †† Department of Biological Sciences, Graduate School of Science, the University of Tokyo, Tokyo, Japan; ‡‡ Institute for Advanced Biosciences, Keio University, Tsuruoka, Japan; §§ PRESTO, Japan Science and Technology Agency, Tokyo, Japan

**Keywords:** Yeast Genome editing, CRISPR-Cas9, Target-AID Base editing, Loss of function screens, Chromosomal fusions

## Abstract

CRISPR-Cas9 loss of function (LOF) and base editing screens are powerful tools in genetics and genomics. Yeast is one of the main models in these fields, but has only recently started to adopt this new toolkit for high throughput experiments. We developed a double selection strategy based on co-selection that increases LOF mutation rates using the Target-AID base editor. We constructed the pDYSCKO vector, which is amenable to high throughput double selection experiments, and show that the improvement in Target-AID efficiency generalizes across loci. Using modeling, we show that this improvement in efficiency provides the required increased in detection power to measure the fitness effects of thousands of mutations in typical yeast pooled screens. We show that double selection can also improve Cas9 mediated LOF rates, but that this multiplex genome editing causes programmable chromosomal translocations at high frequency. This suggests that multiplex LOF editing should be performed with caution and that base-editors could be preferable tools for some screens in yeast. Base editing using double selection is simple and straightforward and provides an alternative to homology directed repair based high throughput variant strain construction methods.

The CRISPR-Cas9 genome editing system has been engineered to create a comprehensive experimental toolkit (Jinek *et al.* 2012; Qi *et al.* 2013; Sander and Joung 2014). One of the principal applications of the system is for gene loss of function (LOF) via DNA repair errors within the coding sequence of a gene after creating targeted double stranded breaks (DSBs). The scalability of this technique allowed for the development of CRISPR-Cas9 LOF experiments at the genome scale (Sanjana *et al.* 2014; Bassett *et al.* 2015; Sidik *et al.* 2016), greatly facilitating systems biology and genomics experiments in models in which they were difficult so far.

Despite yeast being a usual frontrunner in technological developments for systems biology, low CRISPR-Cas9 LOF efficiency in yeast (DiCarlo *et al.* 2013) has so far made the application of this approach impractical. In the same experiments, mutagenesis using CRISPR-Cas9 and donor DNA via the Homology Directed repair pathway (HDR) was shown to be very efficient, but technical challenges prevented large-scale applications until recently. Both the MAGESTIC workflow (Roy *et al.* 2018) and the CHAnGE workflow (Bao *et al.* 2018) rely on mass co-synthesis of the guide sequence and of donor DNA to perform pooled mutagenesis of thousands of variants at the same time in haploid cells. While these methods show remarkable promise, they may be limited for some applications. As synthesis error rates increase with fragment length, these methods require additional experimental resources (*e.g.*: supplementary NGS sequencing) to compensate for the high error rates both in guide sequences and donor DNA. The high toxicity of DSBs (Roy *et al.* 2018) also enriches the pooled populations for cells in which genome editing was not successful, further increasing the amount of sequencing resources required to accurately assess the impact on fitness of engineered mutants. Furthermore, these two workflows require a haploid background, which is rare in wild yeast: many wild and industrial strains are diploid and would therefore require genetic manipulation before they can be used in these high throughput experimental workflows. Therefore, a promising alternative would be to rely on direct base editing that does not depend on double stranded DNA cuts and repair.

Newly developed base editors based on nCAs9 and dCas9 fusions, such as Target-AID (Nishida *et al.* 2016), which induces C to G and C to T changes (with rare C to A), can also be used to create targeted gene LOF by using mutations to generate premature stop codons. While it has been shown that most yeast genes could be theoretically be inactivated using base editing (Billon *et al.* 2017), there has yet to be an experimental assessment of its capabilities as a tool for high throughput screening, even if it has been shown to be highly effective in mammalian cells (Kuscu *et al.* 2017). Target-AID toxicity in yeast has been shown to be far lower than that of Cas9, but its mutagenesis rate remains lower than those of HDR/donor DNA mediated methods.

One of the crucial determinant of large-scale pooled LOF screens is mutagenesis efficiency, which will influence the sequencing resources that have to be invested to detect fitness effects. Accordingly, different approaches have been used to optimize mutagenesis rate. One of them is co-selection, which relies on the selection of cells based on a marker that has mutated alongside the target loci that is positively or negatively selected. This was recently shown to enhance Cas9 edition rates in human cell lines (Agudelo *et al.* 2017) and drosophila (Kane *et al.* 2017), but has not been applied to yeast yet. Co-selection has also be shown to enhance base-editing efficiency in human cell lines (Billon *et al.* 2017). We developed a double selection system in yeast to test if it could increase Target-AID mutagenesis efficiency. We found that this double selection achieves a near 100% mutagenesis rate at some target sites using a vector amenable to genome-wide screening. In early studies, LOF mutagenesis based on DSB repair errors was shown to be highly inefficient in yeast (DiCarlo *et al.* 2013). We revisited these experiments using our double selection strategy and found that this method can be used to increase over a thousand-fold Cas9 LOF editing efficiency, at the cost of inducing frequent chromosomal translocations between the two cut sites.

While it is intuitive that a higher efficiency would allow for more efficient screening as well as the detection of more subtle effects on fitness, it is not clear by how much we need to improve editing efficiency to be able to perform experiments in yeast using CRISPR-Cas9 LOF or base editing so that they would compare in terms of power with those currently performed with the yeast deletion collection. We therefore also developed a statistical model to quantitatively assess the improvement brought by double selection on CRISPR-LOF screens and use it to show that it is highly significant, particularly for fitness effects in the range in which most of gene deletion growth phenotypes are observed across multiple conditions.

## Material and methods

### Experimental procedures

The pDYSCKO vector was built from p426-SNR52p-gRNA.CAN1.Y-SUP4t (Addgene # 43803; all plasmids used in this study are presented in table S1). Directed mutagenesis was used to create silent mutations in the AmpR gene and the *URA3* gene to remove BsaI restriction sites using the oligonucleotides mut_AmpR_For/mut_AmpR_Rev and mut_URA3_For/mut_URA3_Rev (all oligonucleotides used in this study are presented in Table S2). The DYSCKO cassette was synthetized as a gBLOCK (Integrated DNA Technologies, Coralville, USA) and Gibson cloned into the modified backbone amplified with Backbone_For and Backbone_Rev. The g.ADE1 ssDNA oligonucleotide containing the gRNA targeting the *ADE1* gene (as well as the *VPS35* and KanMX4 targeting guides) was amplified by PCR using the gRNA_for and gRNA rev oligonucleotides. This target in *ADE1* was previously used by Nishida *et al.* (Nishida *et al.* 2016) to create LOFs using Target-AID and is also well suited to CRISPR-Cas9 gene disruption. Amplification reactions were pooled and purified using the EZ-10 Column PCR Products Purification Kit (Biobasic, Markham, Canada). Fragment concentration was estimated using a NanoDrop (Thermofisher, Waltham, USA). Guides targeting *POL1*, *SDD3*, *YTM1*, *ARP9*, *RSC3*, *ILV3*, *CDC24* were obtained as dsDNA from Arbor Biosciences (Ann Arbor, USA). The inserts were cloned into pDYSCKO using the Golden Gate Assembly Mix (New England Biolabs, Ipswich, USA) with the following parameters: 1 ul pDYSCKO vector (50 ng/ul), 1 ul insert (∼0.5 ng/ul) using the standard manufacturer assembly reaction conditions, which was incubated for 1 hr at 37° followed by 5 min at 55°. The ligated plasmids were transformed in bacteria and positive clones were confirmed by Sanger sequencing (CHUL sequencing platform, Québec, Canada) using the DYSCKO_rev or DYSCKO_For oligonucleotide. Plasmid p415-Cas9 was obtained from Addgene (#43804). The nCas9-Target-AID plasmid was a generous gift from Dr Keiji Nishida, Kobe University, Kobe, Japan.

All experiments were performed in the haploid *S. cerevisiae* BY4741 strain (*MATa his3Δ1 leu2Δ0 met15Δ0 ura3Δ0*) or diploid BY4743 (*MATa/α his3Δ1/his3Δ1 leu2Δ0/leu2Δ0 LYS2/lys2Δ0 met15Δ0/MET15 ura3Δ0/ura3Δ0*) from Baker Brachmann *et al.* (1998), except for the KanMX4 Cas9 perturbation experiments, which were performed in the ∆*HO* strain from the Yeast Deletion Collection, which was streaked on YPD+G418 (200 µg/ml) after being thawed. A single colony was then used to inoculate a culture for the competent cell protocol.

Synthetic media recipes are detailed in Table S4. All yeast growth occurred at 30° and with shaking in the case of liquid cultures. Competent cells and transformations were performed using standard protocols (Amberg *et al.* 2005), with a two-hour recovery period. Cells were plated on SC-UL and allowed to grow for 48 hr at 30° before the start of mutagenesis experiments.

The same protocol was used for all mutagenesis experiments. Briefly, multiple transformation colonies were used to inoculate a 3 ml SC-UL + 2% glucose culture in which cells were grown for 24 hr. Enough cells to inoculate at 1 OD_600_ a 3ml culture were harvested by centrifugation and placed in SC-UL+5% glycerol for 24 hr. Cas9 or Target-AID expression was then induced by switching the media to 3 ml SC-UL+ 5% galactose for a 12-hour period. Cells were diluted to OD of 0.1 in 3 ml SC-ULR +2% glucose + Canavanine (50 µg/ml) for a 16-hour double selection or 3 ml SC-UL +2% glucose at the same OD as the control condition.

Mutation rates (through loss of function) were assessed by plating cells on SC-ULR + Can and SC-ULR after galactose induction and after recovery with or without double selection at appropriate dilutions. Mutation rate was assessed by calculating the ratio of red (or pink) colonies over total number of colonies. The *ADE1* and *CAN1* alleles of 8 white and 8 red colonies were sequenced to confirm mutations either through Cas9 or Target-AID with CAN1_for, CAN1_rev and ADE1_for, ADE1_rev. LOF rates for the KanMX4 gene measured by resuspending colonies plated on SC-ULR+Can after recovery sterile water, and then spotting 5 µl of the resuspension on YPD and YPD + G418 (200 µg/ml). Growth on YPD but not on YPD+G418 was used to detect LOF of the KanMX4 cassette.

Mutations in essential genes and the non-essential control were also Sanger sequenced (CHUL sequencing platform, Québec, Canada), using the appropriate oligonucleotides (Table S2) and using DNA extracted directly from 100 µl of liquid culture using the Quick DNA method (Lõoke *et al.* 2017). A custom Python script was used to calculate the intensity of mutant *vs.* wild-type peaks on the chromatogram. Briefly, peak locations were extracted from the phred file, and the area under the curve for each nucleotide was calculated in a small window around this position. Intensities were normalized by the total area under the curve at that window, and these values were then used either as is for a heat map or to calculate a mutant/wild-type peak ratio. The titration curve shown in figure S2 was generated by mixing different ratios of cells (based on OD measurements) with known genotypes and then performing a quick DNA extraction. The target site in *ADE1* was then amplified twice and sequenced using the same primers previously described.

The CAN1_For and ADE1_Rev oligonucleotides was used to amplify the *CAN1-ADE1* breakpoint and the resulting amplicon was Sanger sequenced (CHUL sequencing platform, Québec, Canada). Fusions with *VPS35* were engineered using the same approach but with pDYSCKO-*VPS35*. Fusions were detected by PCR using the VPS35-A and CAN1_rev oligonucleotides, and the resulting amplicons were Sanger sequenced (CHUL sequencing platform, Québec, Canada). Sequences were aligned using the MEGA 7 software (Kumar *et al.* 2016).

Chromosomal rearrangements were confirmed by PFGE (Maringele and Lydall 2006). Plugs were prepared using cells from overnight cultures in YPD for BY4741 and SC-ULR + canavanine for mutant strains. Migration time was 27 hr, with a switch time of 60 seconds and no ramping.

### LOF screen model and simulations

In CRISPR-LOF screens, populations of cells are transformed with pools of vectors bearing different gRNAs that are amplified by PCR and used as barcode in a barcode-sequencing competition experiment (Bar-seq) (Shalem *et al.* 2014; Robinson *et al.* 2014), the gRNAs being associated to genes by sequence identity. We modeled this process by adapting haploid selection models, with the added challenge that not all cells with a specific guide will bear a LOF at the target locus because mutation rate is not 100%.

Let $n_{WT}\;$and $n_{BC}$ be respectively the abundance of a control guide and the abundance of a guide targeting a gene which when inactivated has a selection coefficient $S_{mut}$. This mutation is present in a proportion μ, which represents the mutation rate after recovery and thus the relative frequency at the beginning of the competition. After a time t, the abundance ratio of these two barcodes is described by

$$\frac{n_{WT\;(t)}}{n_{BC\;(t)}}=\frac{n_{WT\;0}}{n_{BC\;0}}\cdot \frac{W_{WT\;(t)}}{W_{BC\;(t)}}$$

Were $W_{WT\;(t)}$ is the absolute fitness of the control barcode population at time t and $W_{BC\;(t)}$ the absolute fitness of the barcode associated with the mutation. Because the rate of mutation is not 100% for the strains harboring the barcode (μ<1), a certain proportion of cells (1−μ) will have the same fitness as cells bearing the control barcode so that:

$$W_{BC\;(t)}=\mu \cdot W_{mut\;(t)}+(1-\mu )\cdot W_{WT\;(t)}$$

and

$$\frac{n_{WT\;(t)}}{n_{BC\;(t)}}=\frac{n_{WT\;0}}{n_{BC\;0}}\cdot \frac{W_{WT\;(t)}}{\mu \cdot W_{mut\;(t)}+(1-\mu )\cdot W_{WT\;(t)}}$$

By dividing both the numerator and the denominator of the rightmost factor by $W_{WT\;(t)}$, we obtain

$$\frac{n_{WT\;(t)}}{n_{BC\;(t)}}=\frac{n_{WT\;0}}{n_{BC\;0}}\cdot \frac{1}{\mu \cdot \frac{W_{mut\;(t)}}{W_{WT\;(t)}}+\left(1-\mu \right)}$$

And then to

$$\frac{n_{WT\;(t)}}{n_{BC\;(t)}}=\frac{n_{WT\;0}}{n_{BC\;0}}\cdot \frac{1}{\mu \cdot \frac{W_{mut\;(t)}}{W_{WT\;(t)}}-\mu +1}$$

Because we assume that the growth rates of the mutants and wild-type are constant over time:

$$\frac{W_{mut\;(t)}}{W_{WT\;(t)}}={\left(\frac{W_{mut\;1}}{W_{WT\;1}}\right)}^{t}$$

Given that the selection coefficient $S_{mut}$ is $\left(1-\frac{W_{mut\;1}}{W_{WT\;1}}\right)$ (Hartl 2000) we have

$$\frac{W_{mut\;(t)}}{W_{WT\;(t)}}={\left(1-S_{mut}\right)}^{t}$$

And therefore

$$\frac{n_{WT\;t}}{n_{BC\;t}}=\frac{n_{WT\;0}}{n_{BC\;0}}\cdot \frac{1}{\mu \cdot {\left(1-S_{mut}\right)}^{t}-\mu +1}$$

Which is simplified to

$$\frac{n_{WT\;t}}{n_{BC\;t}}=\frac{n_{WT\;0}}{n_{BC\;0}}\cdot \frac{1}{\mu \cdot ({\left(1-S_{mut}\right)}^{t}-1)+1}$$

If we assume that the control barcode and the mutant are present in the same proportion at the start of the experiment, then $\frac{n_{WT\;0}}{n_{BC\;0}}=1$ and

$$\frac{n_{WT\;t}}{n_{BC\;t}}=\frac{1}{\mu \cdot ({\left(1-S_{mut}\right)}^{t}-1)+1}$$

This gives us a theoretical population ratio at time t for the control barcode over the barcode for a mutant, as a function of the mutagenesis rate and the selection coefficient over a generation. Because we observe these ratios by sequencing the barcode pool at a given depth, the number of reads for each barcode will be influenced by stochastic sampling. If we have k guides in the experiment, and sequence at an estimated depth of d reads on average per guide, and we assume that all barcodes have equivalent abundance at the start, then the number of reads R sequenced for a barcode at t=0 follows

$$R∼B\left(k\cdot d,\;\frac{1}{k}\right)$$

Because of our previous assumptions, this distribution is valid at t=0 for all barcodes regardless of μ or $S_{mut}$. For other values of t, if we assume that the distribution remains equivalent for the control barcodes, then R for a LOF barcoded strain will follow a binomial distribution influenced by the abundance change caused by the LOF of the target allele:

$$R_{MutBC}{}_{\left(t\right)}∼B\left(k\cdot d,\frac{1}{k}\cdot \frac{n_{BC\;(t)}}{n_{WT\;(t)}}\right)$$

Where

$$\frac{n_{WT\;(t)}}{n_{BC\;(t)}}=\frac{1}{\mu \cdot ({\left(1-S_{mut}\right)}^{t}-1)+1}$$

We can therefore obtain simulated sequencing read number distributions as a function of μ, $S_{mut}$, t, k, and d. To assess whether a difference in read count for a barcode is significant, we can test for equality of proportion between a control barcode and the barcode of interest using the chi-square test, with the following table:

**Table:** 

$R_{MutBC\;0}$	$R_{MutBC}{}_{\left(t\right)}$
$R_{wt\;0}$	$R_{wt\;(t)}$

Such experiments include multiple control gRNA that can be pooled in the same category. Most of the time, there are also multiple guides targeting the gene. However, because Target-AID can be used to create specific point mutations that may not all be equivalent to LOF and may not have the same efficiency, it is useful to consider all guides individually. If we set the number of guides per target loci to g, the number of target loci to t, and the number of control c, then the total sequencing depth for a set d changes because:

$$k_{p}=\left(g\cdot t\right)+c$$

Because we expect all controls to be equivalent, then we can assume that

$$R_{ctrl\;0}∼B\left(k_{p}\cdot d,\;\frac{c}{k_{p}}\right),\;\;R_{ctrl\;(t)}∼B\left(k_{p}\cdot d,\;\frac{c}{k_{p}}\right)$$

And for a single target

$$R_{target\;0}∼B\left(k_{p}\cdot d,\;\frac{1}{k_{p}}\right),\;\;R_{target\;(t)}∼B\left(k_{p}\cdot d,\frac{1}{k_{p}}\cdot \frac{n_{BC\;(t)}}{n_{WT\;(t)}}\right)$$

We used this model to simulate a CRISPR-LOF screen with parameters analogous to a Bar-seq screen in yeast. We draw simultaneously a sample from each distribution and test the values for significance over 10 000 iterations. This was done for 100 increments of μ from 0 to 1 and 100 increments of $S_{mut}$ from 0 to 1.

The yeast genome contains ∼6,000 annotated ORFs, of which 1,156 are essential and not useful for a standard LOF screen. Control loci could include intron sequences, pseudogenes or predicted putative non-functional peptides, or often used controls like the HO nuclease. Therefore, to simulate a CRISPR-LOF screen in yeast, the following parameters were used t=4,800 (number of LOF target loci), c=800 (number of control guides), g=8 (number of guides per target loci), d=100 (expected average coverage per guide) and t=26 (number of generations). The CRISPR-Cas9 LOF simulation was performed using a script written in Python 2.7 using the Ipython/Jupyter interactive environment (v4.1.1) (Perez and Granger 2007), Numpy (v1.13.3) (van der Walt *et al.* 2011), Pandas (v0.20.3) (McKinney 2010), Matplotlib (v2.1.0) (Hunter 2007) and SciPy (v0.13.3) (Oliphant 2007). Fitness effects of gene deletions in Bar-seq experiments were retrieved from Qian *et al.* (Qian *et al.* 2012) and pooled across experiments. To generate figure 3b and figure 3c, we used the same model, but used the fitness effects detected in the YP1_replicate of the Qian *et al.* dataset. We then calculated the detection success rate for μ values between 0 and 1 in 0.01 increments, using the same significance threshold and over a thousand iterations. For figure Sx3c, the expected coverage per guide parameter was changed to d=200, but the rest of the parameters remained the same.

### Data availability

The pDYSCKO vector will be deposited on Addgene (http://www.addgene.org/), as well as pDYSCKO-ADE1. The Python 2.7 Packages used are available through in the Python Package Index (https://pypi.python.org/pypi/pip) or in Conda (https://conda.io/docs/). Plasmids used in this study are listed in Table S1 and oligonucleotides in Table S2. A summary of the guides used in this study and their targets is presented in Table S3. Synthetic media recipes are presented in Table S4. Supplemental material available at Figshare: https://doi.org/10.25387/g3.6936683.

## Results and Discussion

**Figure 1:**
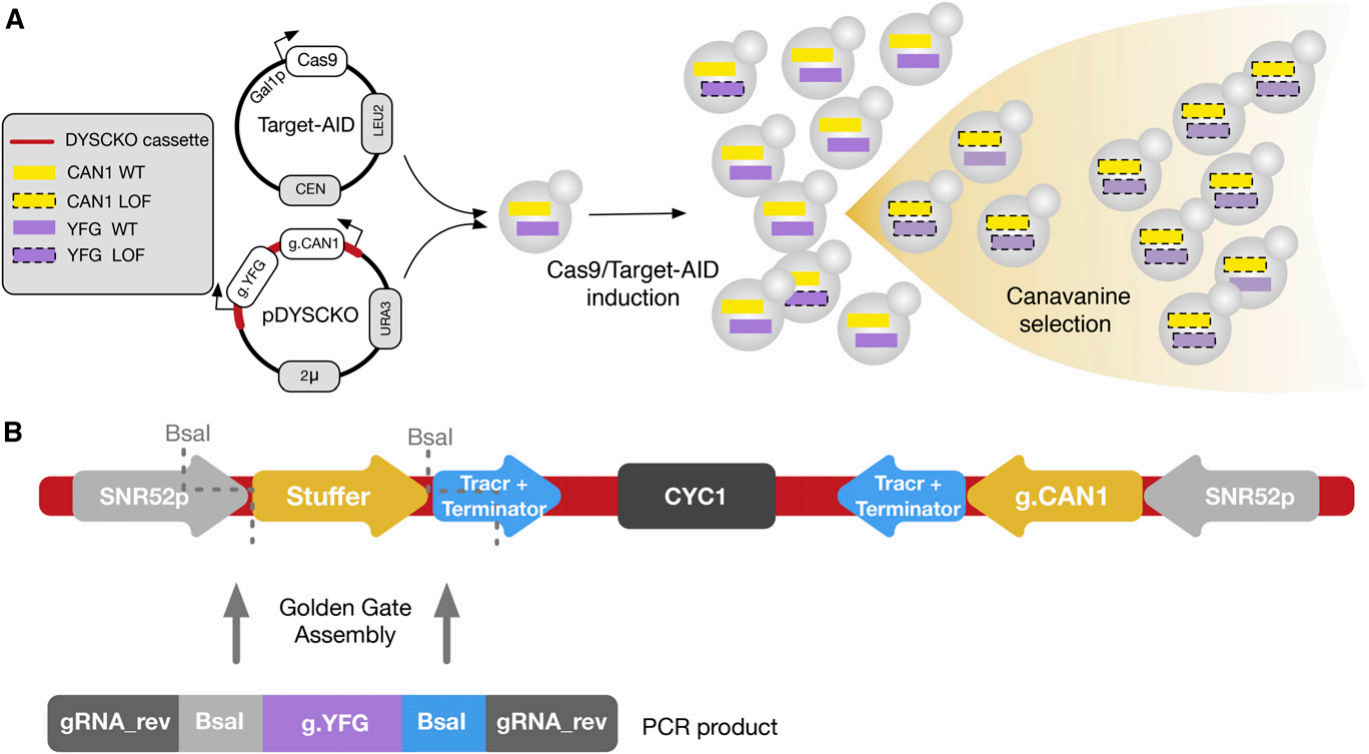
The pDYSCKO-*CAN1* plasmid for LOF double selection. a) Overview of co-selection using the pDYSCKO and the effector plasmid, expressing guide RNAs and the editing enzyme respectively. Only a fraction of cells are successfully edited during induction of the effector enzyme (galactose media), but cells with a LOF at one locus have a greater likelihood of bearing LOFs at both loci. Canavanine selection allows for enrichment for these cells. b) Overall structure of the DYSCKO cassette, which is on a plasmid with standard selection markers (AmpR and URA3). The two symmetrical gRNA expression units use the same promoters and terminators (from SNR52 and SUP4 respectively), insuring their co-expression. The stuffer is a short sequence containing two restriction sites for BsaI that does not match any sequence in the yeast genome. Custom gRNA insertion in the vector is performed through Golden Gate assembly (Engler *et al.* 2008) using a short dsDNA fragment containing the gRNA targeting the sequence of interest (g.YFG).

The double selection system uses the pDYSCKO (Double Yeast Selection CRISPR-KO) plasmid, which encodes for two guide RNAs (gRNA), one targeting the gene of interest (g.YFG) and the other the negative selection market *CAN1* (Figure 1). Another vector is used for galactose induction of the effector enzyme, which can be different variants of Cas9 or Cas9-fusions. LOF of *CAN1* confers resistance to the antibiotic canavanine, which means that canavanine media can be used to enrich the cell population for mutant cells.

**Figure 2:**
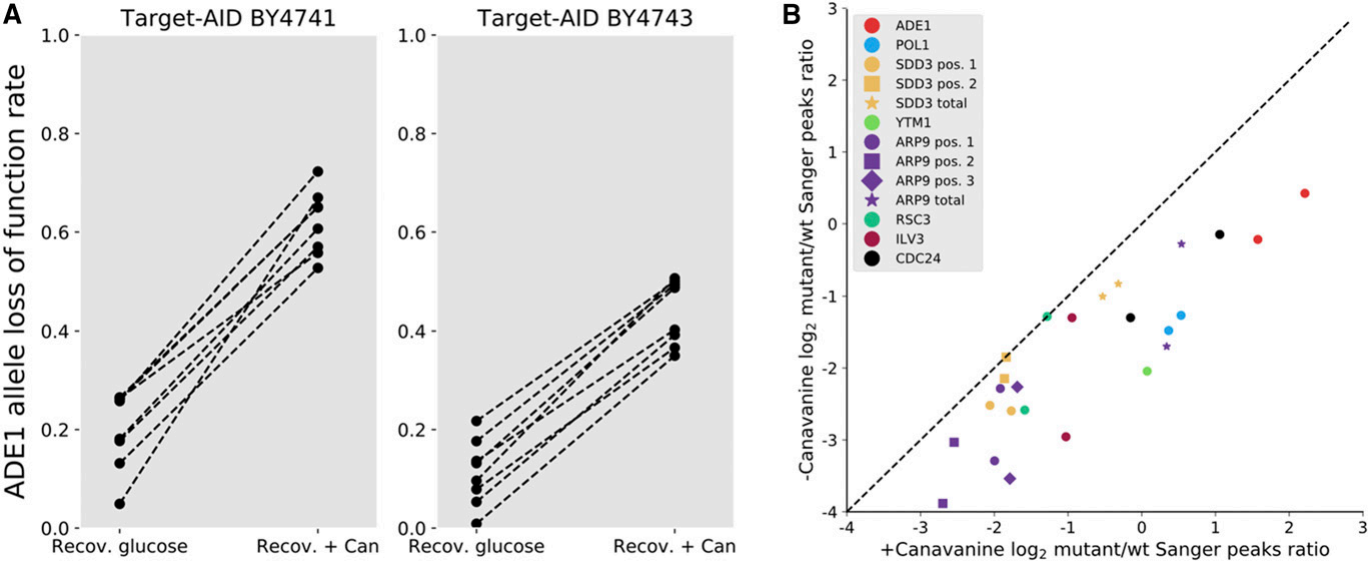
Double selection increases base editing efficiency. a) The same cell cultures were placed in different recovery conditions after induction of Target-AID expression. Recovery in glucose is neutral with respect with the *CAN1* mutant genotype. Recovery in canavanine selects for cells that have LOF mutations at the *CAN1* locus. Selection on the *CAN1* marker increases the representation of *ADE1* LOF colonies. Cells with non-functional *ADE1* alleles accumulate a red metabolic intermediate that gives a characteristic color to colonies, allowing for easy identification of mutants, while mutants without LOF mutations in *ADE1* have a wild-type phenotype. Plating was performed after a 16-hour recovery period. b) Double selection increase mutagenesis rates across multiple target loci. The same cell cultures were placed either in glucose or canavanine media for recovery after the same mutagenesis protocol, with guides targeting different loci (represented by different colors), with mutations at different possible sites (represented by different shapes). Mutation rates were estimated using Sanger sequencing chromatograph peak intensities (see Methods).

We estimated mutation rates using a gRNA that inactivates the *ADE1* gene by a C to G mutation that results in a premature stop codon near the start of the coding sequence. LOF of *ADE1* leads to a red colony phenotype, allowing for direct LOF rate estimation after plating. We compared conditions with and without canavanine double selection after galactose induction. Target-AID LOF rates showed a median fold increase of 3 (median mutation rate: 61%) in haploid cells, and a median fold increase of 4.2 in diploid cells (Figure 2a), showing that our co-selection approach can indeed enrich a cell population for successful edition events. This LOF rate is equivalent to the mutation rate measured at the end of the MAGESTIC workflow (Roy *et al.* 2018), but lower than the estimated rate in the CHAnGE workflow (Bao *et al.* 2018). As Target-AID performs C to T and C to G edition with rare C to A (Nishida *et al.* 2016), and the *ADE1* targeting guide only inactivates *ADE1* if the mutation is C to G (Figure S1), we suspected that the actual mutation rate at the target site might be higher than the LOF rates. Sanger sequencing of white and red colonies confirmed this hypothesis, with all white colonies sequenced bearing a S2L mutation that had no impact on colony phenotype, meaning that the perturbation rate might be higher. If the only goal is perturbation of the target nucleotide (eg.: catalytic site LOF) to change the encoded amino acid, then double selection can achieve near 100% mutagenesis at a target site and thus achieves the same efficiency as HDR/donor DNA methods. To demonstrate the usefulness of double selection, we used it to perform mutagenesis at multiple loci, with a focus on targeting essential genes. To avoid limiting sample size and thus biasing mutation rate assessment, we directly sequenced DNA from cell pools to semi-quantitatively measure allelic frequency in the population. We validated this approach using a titration curve based on variable mutant/wild-type ratios at the *ADE1* locus (Figure S2), which showed that if anything the this method has a tendency to underestimating mutagenesis rates. Mutant/wild-type ratios showed variation in efficiency but systematically show in increased efficiency with the double selection, with on average a twofold improvement (Figure 2c). While none of these guides matched the efficiency of the *ADE1* test, mutational outcomes of essential gene mutagenesis might cause fitness defects or be lethal, which would lower overall efficiency as only unedited cells could survive. For example, mutagenesis using the guide targeting the essential gene *ILV3* can either result in a premature stop codon (C to G, S142*) or a substitution (C to T, S142L), as was the case for the guide targeting *ADE1*. One would therefore expect the C to G mutation to be the most abundant, emulating the pattern observed for the other guide. However, as *ILV3* is essential, this mutation should prove to be lethal. As expected, the C to G mutant is entirely absent after recovery (Figure S3); the observed mutation rate is therefore both dependent on mutagenesis success rate and on the effect on fitness of the mutation.

In a typical CRISPR-LOF screen, pools of gRNA are cloned and transformed in a population of cells. The fate of individual mutants is followed through competition assays in which gRNA sequences serve as barcode to estimate the abundance of each genotype individually by deep sequencing, which is analogous to yeast Bar-seq approaches with the yeast deletion (YKO) collection (Robinson *et al.* 2014). In this type of experiments, one main factor that limits the detection of fitness effects is the depth at which the barcode pool is sequenced. Low mutagenesis efficiency will increase the sequencing resources required to detect fitness effects, potentially making the experiment prohibitively expensive. In the yeast deletion collection, all cells bear a synthetic barcode linked to the gene that has been deleted. This is the same for CRISPR-LOF screens, because the population of cells bearing a specific gRNA are be a mixture of mutants and WTs, which cannot be distinguished from one another by sequencing barcodes alone. The signal observed for a mutant will therefore be a function of the effect of the mutation and the mutation rate within each population bearing the gRNA or associated barcode. A screen using base editors follows the same principles. Lower mutation rates will require increases in sequencing depth to detect the same fitness effects. Using existing data on Bar-seq-based fitness measurements from the YKO collection as a gold standard, we can examine how the improved efficiency of gene editing afforded by the double selection can increase the detection power of fitness effects.

**Figure 3:**
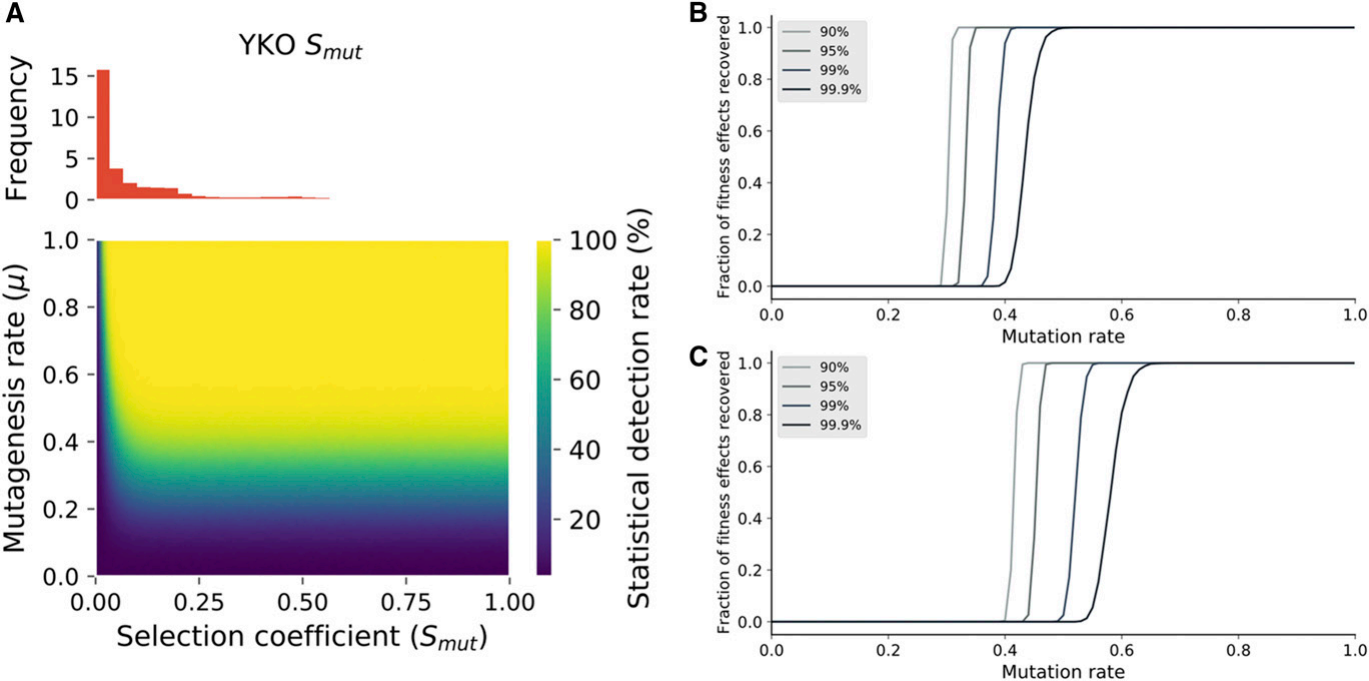
Improvement in mutagenesis rates enhance the power of CRISPR-LOF screens. a) The rate of detection of fitness defects is shown as a function of mutagenesis rate and the selection coefficient of the mutation. Improving the mutation rate above 0.3 dramatically increases the ability to detect growth defects, a requirement in many experiments including genome-wide CRISPR LOF barcode sequencing. The improvement is particularly noticeable for selection coefficient associated to the majority of gene deletion (YKO) mutants in various conditions (Qian *et al.* 2012), as shown in the panel above. b) Fraction of yeast genes for which a significant (*P* < 0.05) fitness effect is detected in our model after LOF based on their YKO fitness coefficients in YPD as a function of mutagenesis rate in a haploid population. The different curves represent different minimal success rates (over a 1000 iterations) used as thresholds to decide whether or not a fitness effect was detected for a given gene at a given mutagenesis rate. c) Same as b), but in a diploid population. Because both alleles of the target must be mutated, higher coverage and higher mutagenesis rates are required to detect the same fitness effects.

Our simulations show that the increased efficiency allows for more sensitive detection of LOF effects (Figure 3a), particularly in the critical zone encompassing fitness effects between 0% and 25%, which are the most frequent effects for the deletion of non-essential genes in yeast. The double selection strategy therefore makes genome-wide screening powerful enough to meet the high standards established by the yeast deletion collection (Figure 3b). For example, at a mutagenesis rate of 0.2, a LOF with a selection coefficient of 0.05 has a 25% chance of being detected, while a rate of 0.6 instead allows it to be detected in over 99% of cases. As visible on the heat map and on the recovery curves, a mutagenesis efficiency above 0.4 appears to be the minimal threshold at which large-scale applications can be considered at a 100x per target depth of sequencing. The model also predicts that the efficiency of Target-AID editing in diploid cells would be high enough to consider large-scale screening applications but would require increasing the coverage twofold (Figure 3c). Of course, such screens often rely on relatively error-prone pooled synthesis of the gRNA sequences, meaning that a significant fraction of cells within the population can bear a guide that cannot mutate the intended target. As such, additional sequencing resources are needed to compensate, something for which our model does not account. Because these experiments would be feasible in diploid cells and because our plasmid selection markers on our vectors can easily be changed to antibiotic resistance cassettes for G418 (Wach *et al.* 1994), Nourseothricin or Hygromycin B (Goldstein and McCusker 1999), double selection after Target-AID could allow for high throughput marker less gene disruption in non-standard laboratory and wild strains.

**Figure 4:**
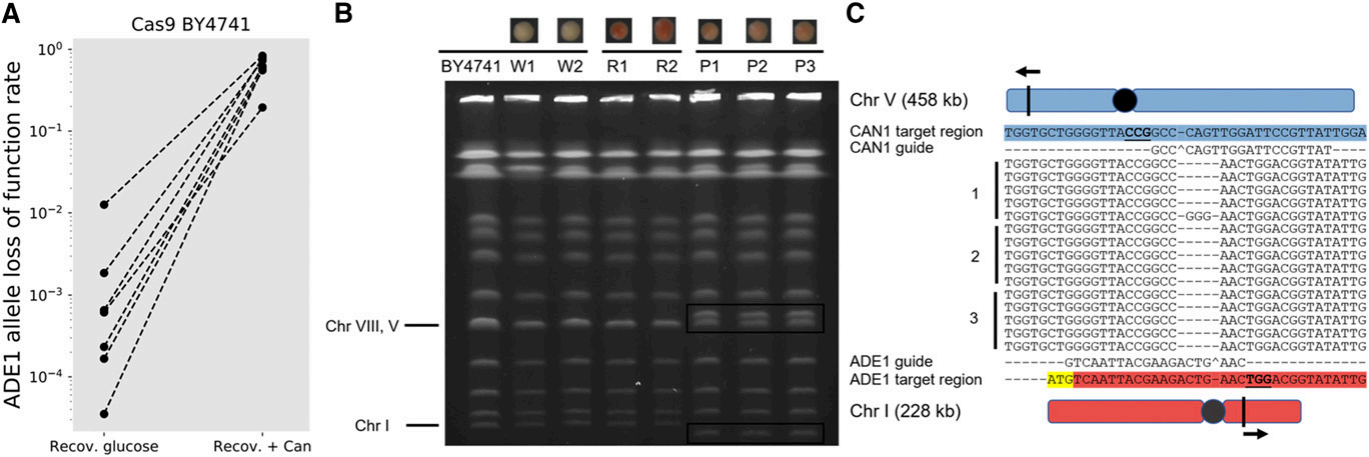
Multiplex CRISPR-Cas9 causes chromosomal rearrangements in yeast. a) Double selection increases LOF rates by Cas9 mutagenesis in yeast. The same experiment as in figure 2a was performed. Red and pink colonies were considered to bear a LOF. b) Chromosomal migration patterns of the different canavanine resistant mutants generated using Cas9. Mutants without LOF mutations in *ADE1* have the same phenotype as the wild-type strain. Colonies with a distinct pink color show chromosomal fusions. *CAN1* only LOF (W1 and W2) and red *CAN1*, *ADE1* LOF mutants show a chromosomal migration pattern that is comparable to the wild-type. Pink colonies from independent mutagenesis experiments show altered length for chromosomes I and chromosomes V, consistent with a reciprocal translocation centered on the predicted Cas9 cut sites. b) Breakpoint sequencing of 5 pink colonies from 3 independent mutagenesis experiments confirms PFGE patterns.

After observing that double selection could enhance base editing efficiency, we hypothesized it could also help enhance LOF rates by Cas9 via errors in DSB repairs. We therefore performed the same assay as for Target-AID mutagenesis but using Cas9 as the effector enzyme instead. Double selection increased the Cas9 LOF rates over 1200-fold to a median mutation rate of 62% (Figure 4a). Experiments using multiple other guides targeting the KanMX4 resistance cassette showed LOF rates as high as 94% and as low as 34% (n = 3 for each guide, 32 colonies tested per replicate), consistent with the known variance in guide effectiveness depending on sequence context.

Most importantly, our experiment also revealed that multiplexed gene targeting for double-strand break with CRISPR-Cas9 in yeast has undesirable side effects. We recurrently observed colonies exhibiting an intermediate phenotype for *ADE1* LOF (median rate: 18%), with a color pattern slightly different from the standard red *ade1* phenotype. We found that these cells harbored a chromosomal rearrangement that results into a *CAN1-ADE1* fusion. We first identified these fusions by PCR and Sanger sequencing and confirmed that they affect chromosome size by PFGE (Figure 4b, 4c). Changing the DYSCKO target locus from *ADE1* to *VPS35* produced a different fusion but at a similar rate (Figure S4), showing that these fusions are likely programmable and generalizable. The observation that multiplex genome editing that does not rely on HDR integration of donor DNA causes chromosomal rearrangements at relatively high frequency is worrisome because many recent multiplex mutagenesis workflow rely on PCR based deep sequencing of the target allele to measure success rates (*i.e.*, CRISPR-Seq (Tothova *et al.* 2017)) of genome editing at multiple loci. This method cannot detect chromosomal rearrangements, and as such the events may have gone unnoticed in many experiments so far. This ability of the CRISPR-Cas9 system has been known for some time (Maddalo *et al.* 2014). It should be noted that the high occurrence rate of chromosomal translocations might be specific to yeast, but efforts should be made to assess it in other models, even if this it made harder by the absence of an easily detectable phenotype. Recently, cleavage by Cas9 was shown to result in large deletions and chromosomal rearrangements at the target site in mouse and human stem cells (Kosicki *et al.* 2018). Our findings may therefore be generalizable, with features specific to each cell type.

As we have yet to observe any case of rearrangements when using Target-AID, base editors might be safer choices in most cases when attempting multiplex LOF mutagenesis. Base editor have already been shown to be viable alternatives to Cas9 based LOFs for high throughput screening based on mutations that creating stop codons (Kuscu *et al.* 2017). One possible tradeoff with these editors is that some of the induced changes may lead to silent substitutions, such as the ones we observed here, which effectively limit the effective LOF rate. Our approach relies on the *CAN1* gene that can be used for negative selection against non-mutated cells. This marker has been used in many large-scale and systematic experiments, for instance synthetic gene arrays (SGA) (Tong *et al.* 2001), which means that the edited strains are compatible in terms of genotype with a broad range of methods routinely used by the yeast community. If needed, other negatively selectable markers could be exploited, for instance *LYP1*, *FCY1* and *URA3*. The development of other base editors, for instance from A-T to G-C (Gaudelli *et al.* 2017), will also offer new possibilities in terms of producing loss of function mutations without DNA cleavage. Base editing capabilities will also increase as new Type II CRISPR/Cas9 are adapted (Verwaal *et al.* 2018) or engineered for added versatility (Kleinstiver *et al.* 2015), increasing their versatility. Base editing in yeast can become a powerful tool for yeast systems biology that can complement existing high throughput approaches while opening new experimental avenues.
